# Supplementary figures and images for: Development and evaluation of a live birth prediction model for evaluating human blastocysts from a retrospective study
Source: eLife. 2023 Feb 22;12:e83662. doi: 10.7554/eLife.83662 (PMC10069866; doi:10.7554/eLife.83662)

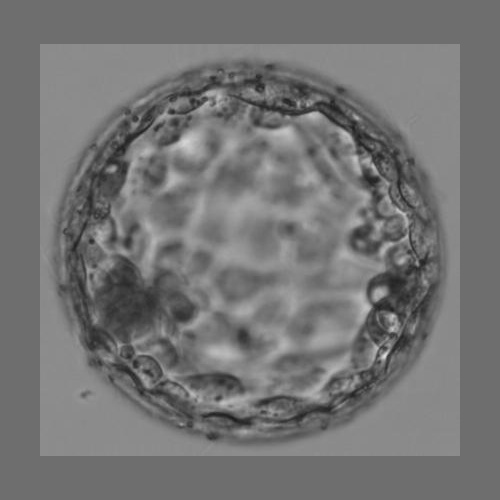

Supplement: Figure 4—source data 1. [file elife-83662-fig4-data1.zip › Figure 4-Source Data 1/1_focus_0.jpg]

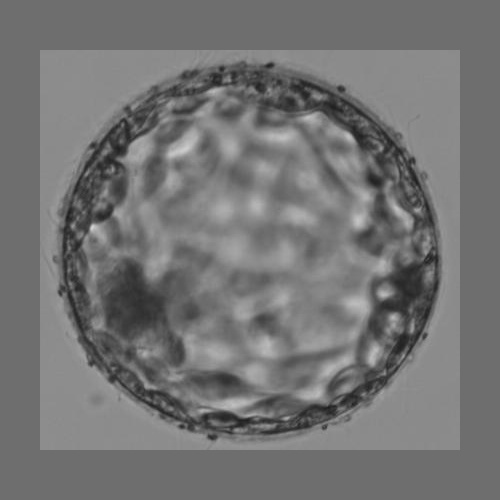

Supplement: Figure 4—source data 1. [file elife-83662-fig4-data1.zip › Figure 4-Source Data 1/1_focus_1.jpg]

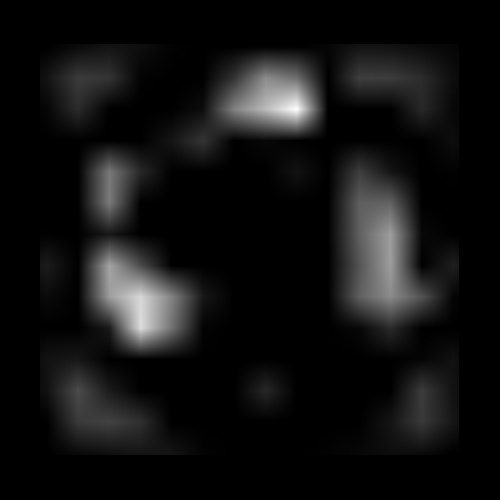

Supplement: Figure 4—source data 1. [file elife-83662-fig4-data1.zip › Figure 4-Source Data 1/1_image_and_clinical_weight.jpg]

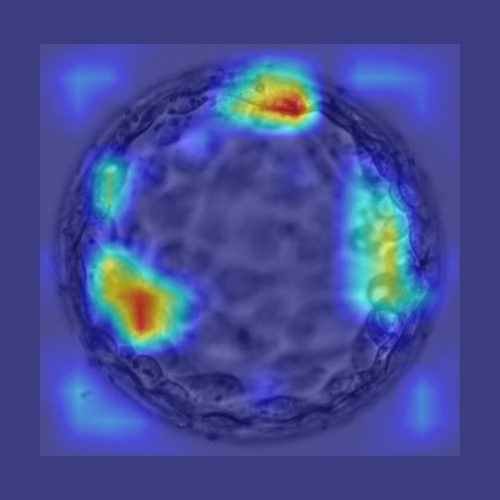

Supplement: Figure 4—source data 1. [file elife-83662-fig4-data1.zip › Figure 4-Source Data 1/1_image_clinical_heatmap.jpg]

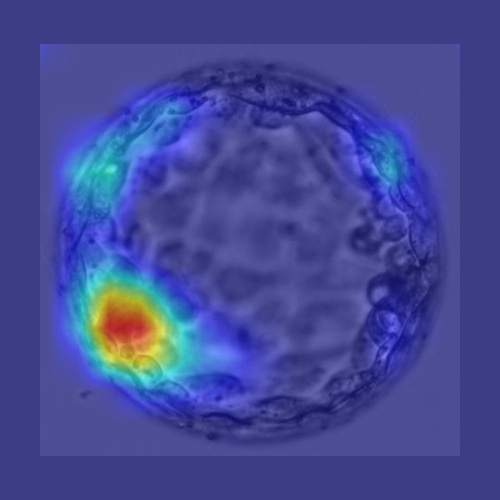

Supplement: Figure 4—source data 1. [file elife-83662-fig4-data1.zip › Figure 4-Source Data 1/1_image_heatmap.jpg]

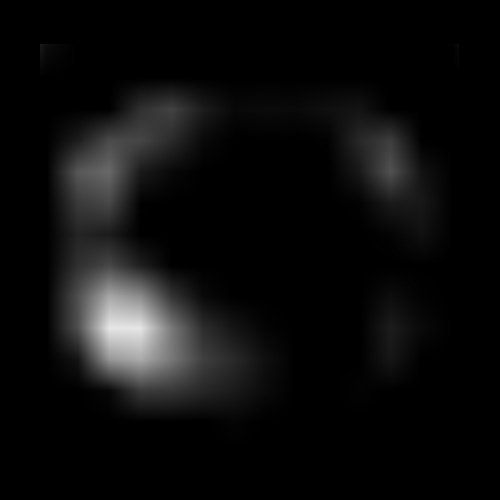

Supplement: Figure 4—source data 1. [file elife-83662-fig4-data1.zip › Figure 4-Source Data 1/1_image_weight.jpg]

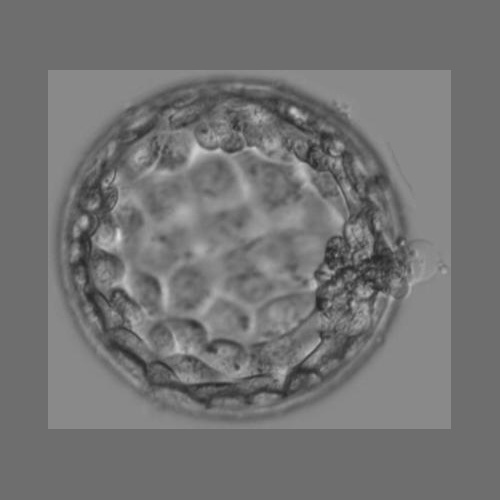

Supplement: Figure 4—source data 1. [file elife-83662-fig4-data1.zip › Figure 4-Source Data 1/2_focus_0.jpg]

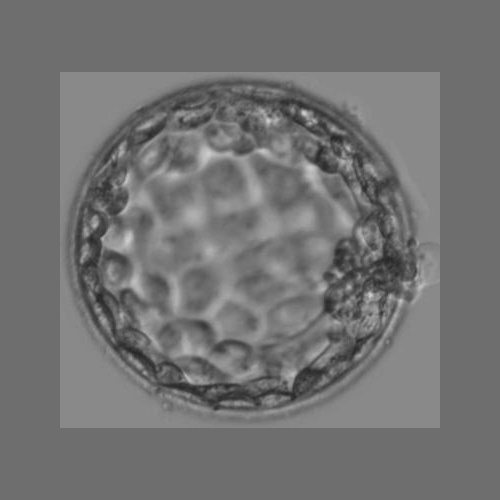

Supplement: Figure 4—source data 1. [file elife-83662-fig4-data1.zip › Figure 4-Source Data 1/2_focus_1.jpg]

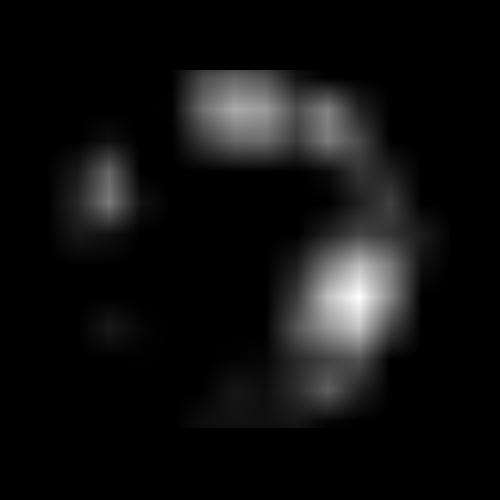

Supplement: Figure 4—source data 1. [file elife-83662-fig4-data1.zip › Figure 4-Source Data 1/2_image_and_clinical_weight.jpg]

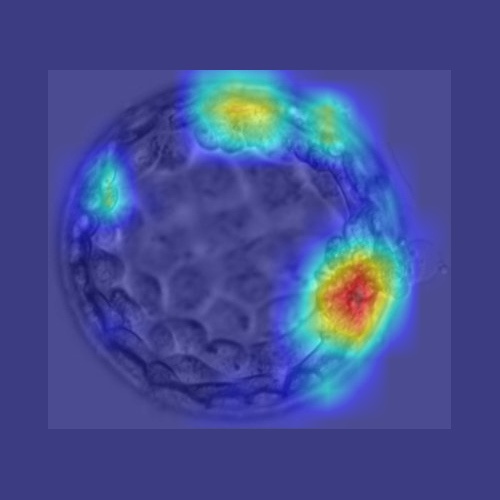

Supplement: Figure 4—source data 1. [file elife-83662-fig4-data1.zip › Figure 4-Source Data 1/2_image_clinical_heatmap.jpg]

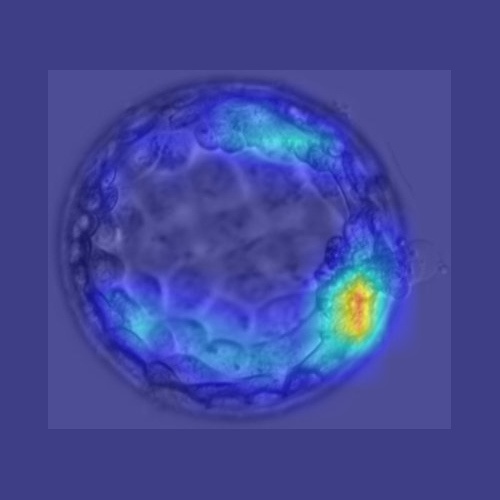

Supplement: Figure 4—source data 1. [file elife-83662-fig4-data1.zip › Figure 4-Source Data 1/2_image_heatmap.jpg]

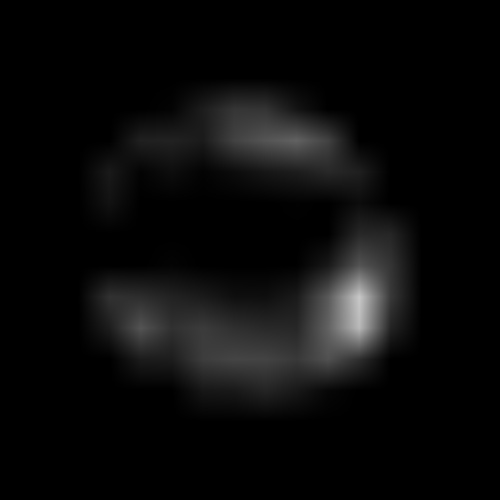

Supplement: Figure 4—source data 1. [file elife-83662-fig4-data1.zip › Figure 4-Source Data 1/2_image_weight.jpg]

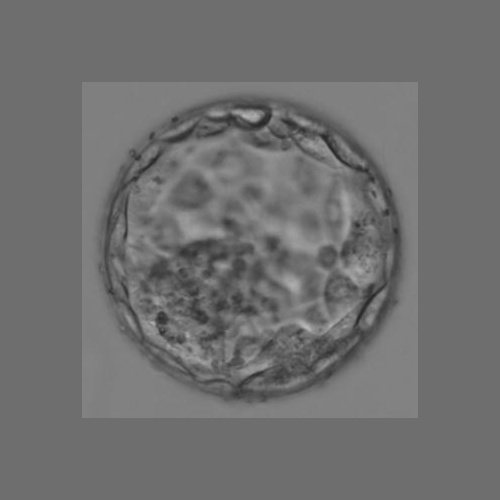

Supplement: Figure 4—source data 1. [file elife-83662-fig4-data1.zip › Figure 4-Source Data 1/3_focus_0.jpg]

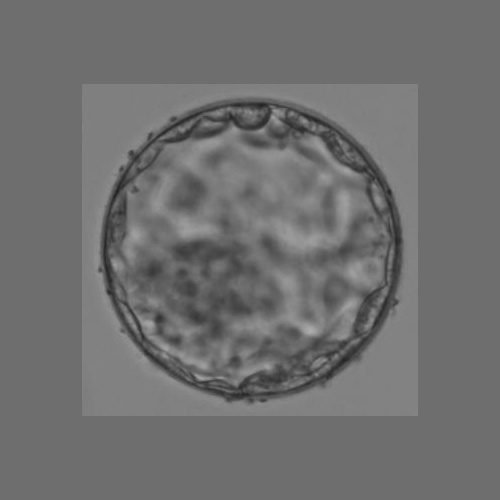

Supplement: Figure 4—source data 1. [file elife-83662-fig4-data1.zip › Figure 4-Source Data 1/3_focus_1.jpg]

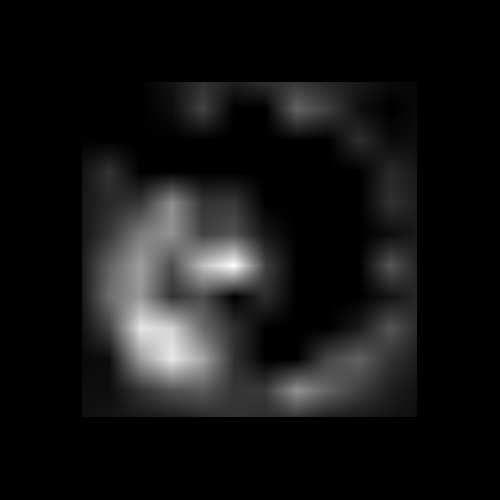

Supplement: Figure 4—source data 1. [file elife-83662-fig4-data1.zip › Figure 4-Source Data 1/3_image_and_clinical_weight.jpg]

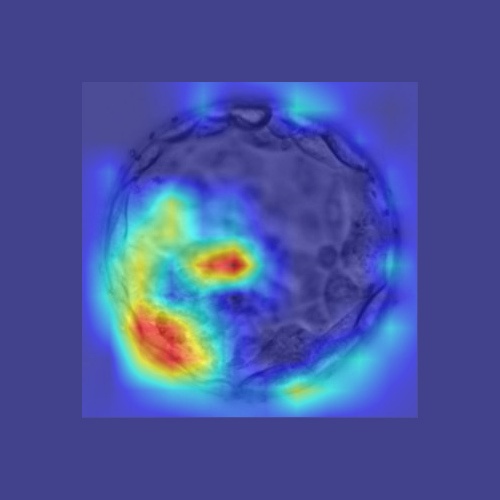

Supplement: Figure 4—source data 1. [file elife-83662-fig4-data1.zip › Figure 4-Source Data 1/3_image_clinical_heatmap.jpg]

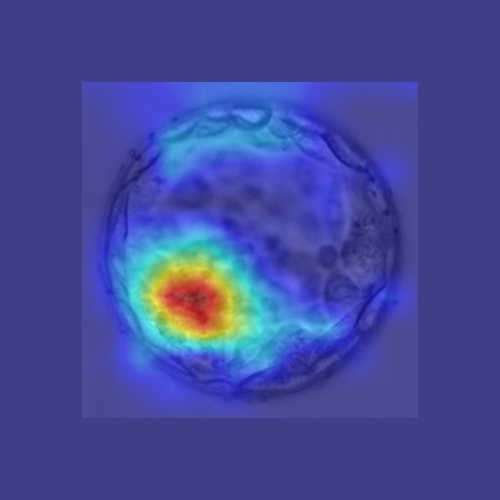

Supplement: Figure 4—source data 1. [file elife-83662-fig4-data1.zip › Figure 4-Source Data 1/3_image_heatmap.jpg]

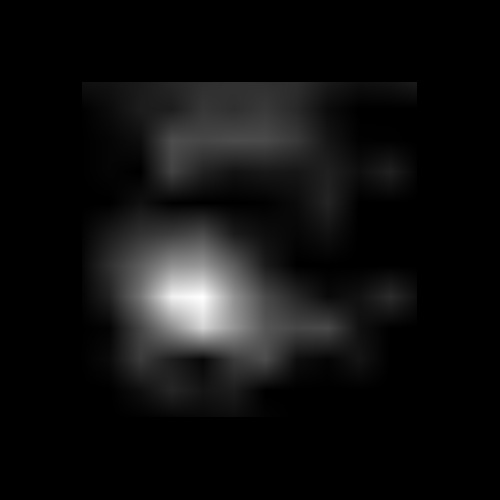

Supplement: Figure 4—source data 1. [file elife-83662-fig4-data1.zip › Figure 4-Source Data 1/3_image_weight.jpg]

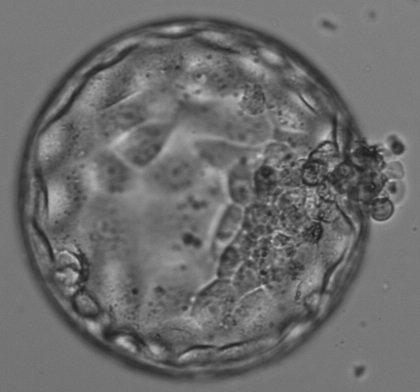

Supplement: Figure 4—figure supplement 1—source code 1. [file elife-83662-fig4-figsupp1-code1.zip › Figure-4-Supplement-1-Code/img_to_be_padded.jpg]

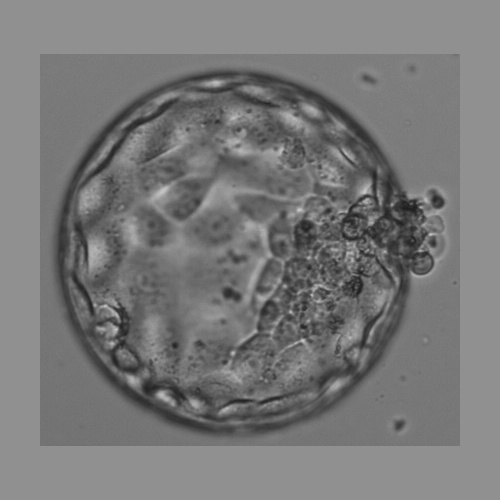

Supplement: Figure 4—figure supplement 1—source code 1. [file elife-83662-fig4-figsupp1-code1.zip › Figure-4-Supplement-1-Code/padded_img.jpg]
