# Supplementary material for: Development and evaluation of a live birth prediction model for evaluating human blastocysts from a retrospective study
Source: eLife. 2023 Feb 22;12:e83662. doi: 10.7554/eLife.83662 (PMC10069866; doi:10.7554/eLife.83662)
Supplement: Supplementary file 1. [file elife-83662-supp1.docx]

Supplementary file 1

Supplementary Table Ⅰ. P-value analysis result of 103 patient couple's clinical features.

Fifty-one out of the 103 clinical features, highlighted in bold text, were identified to have statistical significance between blastocysts with positive and negative live birth outcomes. Y: yes, N: No.

| ID | Feature name | P-value ≤ 0.05 |  | ID | Feature name | P-value ≤ 0.05 |
| --- | --- | --- | --- | --- | --- | --- |
| Categorial features | | |  | 32 | Clomiphene citrate | N |
|  |  |  |  | 33 | Progesterone | N |
| **1** | **Infertility type** | **Y** |  | **34** | **Mild stimulation** | **Y** |
| 2 | Tubal obstruction | N |  | **35** | **GnRH antagonists** | **Y** |
| **3** | **Ovulatory dysfunction** | **Y** |  | 36 | Letrozole | N |
| **4** | **Endometriosis** | **Y** |  | **37** | **Short effect long scheme** | **Y** |
| 5 | Oligozoospermia | N |  | **38** | **Natural cycle** | **Y** |
| 6 | Asthenospermia | N |  | 39 | Dydrogesterone | N |
| 7 | Oligoasthenozoospermia | N |  | 40 | Short down-regulated | N |
| 8 | Teratozoospermia | N |  | 41 | Ultra-short down-regulated | N |
| 9 | Maternal immune infertility | N |  | **42** | **Long down-regulated** | **Y** |
| 10 | Obstructive azoospermia | N |  | **43** | **Ultra-long down-regulated** | **Y** |
| **11** | **Azoospermia** | **Y** |  | 44 | Ultra-ultra-long down-regulated | N |
| 12 | Abnormal acrosome | N |  |  | Insemination method |  |
| 13 | Paternal chromosome abnormalities | N |  | **45** | **IVF** | **Y** |
| **14** | **Ovarian hyperstimulation syndrome** | **Y** |  | 46 | ICSI | N |
| **15** | **Poor endometrial receptivity** | **Y** |  | 47 | Combined | N |
| 16 | Maternal single-gene genetic disease | N |  |  | Sperm source |  |
|  | Maternal blood type |  |  | **48** | **Frozen semen** | **Y** |
| 17 | A | N |  | **49** | **Fresn semen** | **Y** |
| 18 | AB | N |  | **50** | **Testicular sperm aspiration** | **Y** |
| 19 | B | N |  | **51** | **Percutaneous epidydimal sperm aspiration** | **Y** |
| 20 | O | N |  |  | Sperm preparation techniques |  |
|  | Paternal blood type |  |  | 52 | Swim up | N |
| 21 | A | N |  | 53 | Hypo-osmotic swelling | N |
| **22** | **AB** | **Y** |  | **54** | **Density gradient centrifugation** | **Y** |
| 23 | B | N |  | 55 | Wash | N |
| 24 | O | N |  | 56 | Wash and swim up | N |
|  | Endometrium (EM) pattern before transfer |  |  | Numerical features | | |
| **25** | **A** | **Y** |  |  |  |  |
| 26 | B | N |  | **57** | **Maternal age** | **Y** |
| 27 | C | N |  | **58** | **Maternal body mass index** | **Y** |
|  | EM pattern on HCG day |  |  | **59** | **Infertility duration** | **Y** |
| **28** | **A** | **Y** |  | **60** | **Number of OS cycles** | **Y** |
| **29** | **B** | **Y** |  | **61** | **EM thickness before transfer** | **Y** |
| **30** | **C** | **Y** |  | **62** | **Prolactin before transfer** | **Y** |
|  | Ovarian stimulation (OS) protocols |  |  | **63** | **Progesterone before transfer** | **Y** |
| 31 | GnRH agonist long protocol | N |  | **64** | **Estradiol before transfer** | **Y** |

| ID | Feature name | P value ≤ 0.05 |  | ID | Feature name | P value ≤ 0.05 |
| --- | --- | --- | --- | --- | --- | --- |
| Continued | | |  | 102 | Ratio of grade D sperms before processing | N |
|  |  |  |  | 103 | Ratio of grade D sperms after processing | N |
| 65 | Number of OS days | N |  |  | | |
| **66** | **Days from OS to HCG day** | **Y** |  |  |  |  |
| **67** | **Days from OS to ovum pick-up** | **Y** |  |  |  |  |
| **68** | **EM thickness on HCG day** | **Y** |  |  |  |  |
| **69** | **Prolactin on HCG day** | **Y** |  |  |  |  |
| **70** | **Progesterone on HCG day** | **Y** |  |  |  |  |
| **71** | **Estradiol on HCG day** | **Y** |  |  |  |  |
| **72** | **Luteinising hormone on HCG day** | **Y** |  |  |  |  |
| **73** | **Left antral follicle count** | **Y** |  |  |  |  |
| **74** | **Right antral follicle count** | **Y** |  |  |  |  |
| **75** | **Total antral follicle count** | **Y** |  |  |  |  |
| **76** | **Follicle stimulating hormone on day 3 after period** | **Y** |  |  |  |  |
| **77** | **Luteinising hormone on day 3 after period** | **Y** |  |  |  |  |
| 78 | Estradiol on day 3 after period | N |  |  |  |  |
| 79 | Prolactin on day 3 after period | N |  |  |  |  |
| 80 | Progesterone on day 3 after period | N |  |  |  |  |
| 81 | Total testosterone on day 3 after period | N |  |  |  |  |
| 82 | Free triiodothyronine on day 3 after period | N |  |  |  |  |
| **83** | **Free thyroxine on day 3 after period** | **Y** |  |  |  |  |
| **84** | **Anti-Müllerian hormone on day 3 after period** | **Y** |  |  |  |  |
| 85 | Cancer antigen 125 | N |  |  |  |  |
| 86 | Blood sugar | N |  |  |  |  |
| 87 | Thyroid-stimulating hormone on day 3 after period | N |  |  |  |  |
| **88** | **Ratio of progesterone to estradiol on HCG day** | **Y** |  |  |  |  |
| **89** | **Retrieved oocyte number** | **Y** |  |  |  |  |
| 90 | Days of abstinence | N |  |  |  |  |
| **91** | **Day of blastocyst transfer** | **Y** |  |  |  |  |
| **92** | **Semen volume before processing** | **Y** |  |  |  |  |
| 93 | Semen volume after processing | N |  |  |  |  |
| 94 | Sperm density before processing | N |  |  |  |  |
| 95 | Sperm density after processing | N |  |  |  |  |
| 96 | Ratio of grade A sperms before processing | N |  |  |  |  |
| **97** | **Ratio of grade A sperms after processing** | **Y** |  |  |  |  |
| 98 | Ratio of grade B sperms before processing | N |  |  |  |  |
| **99** | **Ratio of grade B sperms after processing** | **Y** |  |  |  |  |
| 100 | Ratio of grade C sperms before processing | N |  |  |  |  |
| 101 | Ratio of grade C sperms after processing | N |  |  |  |  |
